# Neuropsychiatric Symptoms in Pediatric Chronic Pain and Outcome of Acceptance and Commitment Therapy

**DOI:** 10.3389/fpsyg.2021.576943

**Published:** 2021-04-09

**Authors:** Leonie J. T. Balter, Camilla Wiwe Lipsker, Rikard K. Wicksell, Mats Lekander

**Affiliations:** ^1^Department of Psychology, Stress Research Institute, Stockholm University, Stockholm, Sweden; ^2^Division of Psychology, Department of Clinical Neuroscience, Karolinska Institutet, Stockholm, Sweden; ^3^Functional Area Medical Psychology, Functional Unit Behavior Medicine, Karolinska University Hospital, Stockholm, Sweden

**Keywords:** pediatric chronic pain, autism spectrum disorder, attention-deficit hyperactivity disorder, acceptance and commitment therapy, socioemotional functioning

## Abstract

Considerable heterogeneity among pediatric chronic pain patients may at least partially explain the variability seen in the response to behavioral therapies. The current study tested whether autistic traits and attention-deficit/hyperactivity disorder (ADHD) symptoms in a clinical sample of children and adolescents with chronic pain are associated with socioemotional and functional impairments and response to acceptance and commitment therapy (ACT) treatment, which has increased psychological flexibility as its core target for coping with pain and pain-related distress. Children and adolescents aged 8–18 years (*N* = 47) were recruited. Patients and their parents completed questionnaires pre- and post-ACT of 17 sessions. Correlational analyses and mixed-effects models were used to assess the role of autistic traits and ADHD symptoms in pretreatment functioning and ACT-treatment response. Outcome variables were degree to which pain interfered with daily activities (i.e., pain interference, sleep, and physical and school functioning), socioemotional functioning (i.e., depressive symptoms, emotional, and social functioning), psychological inflexibility, and pain intensity. Autistic traits and ADHD symptoms, pain frequency, and pain duration were measured at pretreatment only. Higher autistic traits were associated with greater pain interference, higher depression, and greater psychological inflexibility. Higher ADHD symptomatology was associated with greater pretreatment pain interference, lower emotional functioning, greater depression, and longer duration of pain. Across patients, all outcome variables, except for sleep disturbances and school functioning, significantly improved from pre- to post-ACT. Higher autistic traits were associated with greater pre- to post-ACT improvements in emotional functioning and sleep disturbance and non-significant improvements in pain interference. ADHD symptomatology was not associated with treatment outcome. The current results showed that neuropsychiatric symptoms in pediatric chronic pain patients are associated with lower functioning, particularly pain interfering with daily life and lower socioemotional functioning. The results suggest that not only pediatric chronic pain patients low in neuropsychiatric symptoms may benefit from ACT, but also those high in autism traits and ADHD symptoms. With the present results in mind, pediatric chronic pain patients higher in autistic traits may actually derive extra benefit from ACT. Future research could assess whether increased psychological flexibility, the core focus of ACT, enabled those higher in autism traits to cope relatively better with pain-related distress and thus to gain more from the treatment, as compared to those lower in autism traits. Moreover, to address specific effects of ACT, inclusion of an appropriate control group is key.

## Introduction

Pediatric chronic pain is often accompanied by mental health comorbidities such as depressive and anxiety symptoms, which contribute to lower quality of life and worse daily functioning (i.e., school absenteeism, social withdrawal). Such comorbid problems posit additional physical and emotional burdens for patients and their families (Gold et al., 2009; Miller and Cano, 2009; Hoftun et al., 2011). Chronic pain in childhood and adolescence further incurs a high risk for development of widespread pain, psychological comorbidities, and lower functional status later in life (Hassett et al., 2013). Hence, there is great need for interventions that effectively improve functioning in pediatric chronic pain patients.

While nearly half of the pediatric chronic pain patients suffer from comorbid mental health disorders including mood and anxiety disorders (Vinall et al., 2016; Fisher et al., 2018), neuropsychiatric disorders such as autism and attention-deficit/hyperactivity disorder (ADHD) seem to go unrecognized (Lipsker et al., 2018; Low Kapalu et al., 2018). Lipsker et al. (2018) recently showed that clinically significant levels of autism traits and/or ADHD symptoms were indicated in more than a quarter of children and adolescents with chronic pain. Similarly, chronic pain occurs in a large proportion of individuals diagnosed with clinical autism and/or ADHD (Asztély et al., 2019; Whitney and Shapiro, 2019).

Meta-analyses have shown that psychological therapies, particularly those based on cognitive behavior therapy (CBT), can improve functioning in chronic pain in children, adolescents, and adults (Eccleston et al., 2002; Palermo et al., 2010; Veehof et al., 2011, 2016; Fisher et al., 2014; Hann and McCracken, 2014; Öst, 2014; Hughes et al., 2017). However, little is known about how comorbid neuropsychiatric problems may be associated with treatment outcome (Coffelt et al., 2013; Harrison et al., 2014; Liossi and Howard, 2016; Vinall et al., 2016). Studies suggest that comorbid emotional distress predicts acceptance and commitment therapy (ACT) response, but the results are inconsistent, and the direction of the association has varied between studies (Gilpin et al., 2017). Higher pretreatment psychological distress in different types of chronic pain has been associated with both greater and poorer improvements in functioning and pain interference after psychological treatment (Zautra et al., 2008; Trompetter et al., 2016; Tseli et al., 2019). Also no relationship between distress and psychological treatment outcome has been reported (Broderick et al., 2016; Wetherell et al., 2016). Because distress is a broad umbrella term for adversities, more specific characterization of comorbid problems is warranted to understand for whom treatment is likely to work and to better tailor treatments to individual needs. Moreover, most of these studies that addressed the role of emotional distress in ACT response have been conducted with adults, leaving largely unexplored the relationship between emotional distress and ACT outcome in pediatric chronic pain.

Poor socioemotional functioning is a characteristic of many of the psychiatric disorders comorbid to chronic pain (i.e., autism, ADHD, depression, and anxiety) (Liossi and Howard, 2016; Smith and White, 2020). Psychological interventions targeting socioemotional functioning may thus be specifically beneficial for patients with greater levels of autism traits and ADHD symptoms. ACT, a development within CBT, is such a psychological treatment for chronic pain with potential to improve emotional distress and affective symptoms (i.e., reduce anxiety or depressive symptoms) and to reduce pain interfering with daily life (Veehof et al., 2011, 2016; Hann and McCracken, 2014; Öst, 2014; Pahnke et al., 2014). ACT aims to improve daily functioning by strengthening active and accommodative coping strategies through increasing psychological flexibility. Patients are encouraged to engage in behaviors that are in line with personal and meaningful values. In this process, acceptance of what cannot be changed (e.g., pain) and recognition of the things that can be changed, such as behaviors that serve valued ends, are emphasized. By helping the patient to recognize and acknowledge negative thoughts (e.g., “going to school will make my pain worse”), the therapist helps the patient to distance oneself from the thoughts rather than by discussing whether or not the thoughts are correct (Hayes and Wilson, 1994). Increased psychological flexibility can thus serve as a coping mechanism to improve dealing with pain and pain-related distress (Vowles et al., 2014b).

The empirical support for ACT in adults with unspecific chronic pain is considered as strong (Vowles et al., 2014a). A growing body of evidence suggests that ACT may have a similarly positive impact on children and adolescents with chronic pain (e.g., Kanstrup et al., 2016), although this notion is preliminary due to a limited number of studies performed in pediatric chronic pain patients. For example, in two clinical pilot studies, ACT improved adolescent functioning (Kanstrup et al., 2016; Martin et al., 2016). A case example of an adolescent with chronic pain, as well as a few studies including children or adolescents, likewise documented improvements in daily life functioning (e.g., school attendance) following ACT (Wicksell et al., 2005; Gauntlett-Gilbert et al., 2013; Ghomian and Shairi, 2014). However, studies addressing the effect of ACT on socioemotional outcomes, such as depressive symptoms, emotional and social functioning, and sleep, are limited in pediatric chronic pain patients (reviewed in Fisher et al., 2014). This is of particular interest in the context of neurodevelopmental disorders such as autism disorder and ADHD, which appear to be comorbid to pediatric chronic pain to a larger extent than in healthy populations and have socioemotional dysfunction as a core feature (Lipsker et al., 2018). In a study of Pahnke et al. (2014), involving a sample of 13–21-year-old high-functioning students with autism, increased prosocial behavior and reduced stress, hyperactivity, and emotional distress were observed after ACT treatment. Individuals with autism often exhibit rigidity and a need for rule-governed behaviors (Leekam et al., 2011), which has been suggested to contribute to chronic pain (Beeckman et al., 2019). Therefore, ACT's focus on strengthening coping strategies may further enhance its effects in pediatric chronic pain patients high in traits and symptoms of neurodevelopmental disorders (Simons et al., 2008; Wicksell et al., 2011). Furthermore, executive dysfunctions, such as cognitive inflexibility and attentional deficits, are prevalent in autism and ADHD, although with a different focus between the disorders, and have likewise been linked to how patients cope with pain (Corbett et al., 2009; Berryman et al., 2014; Craig et al., 2016). The biopsychosocial model of Compas and Boyer (2001) highlights the importance of attentional processes in coping with pain. Shifting focus away from pain and sustaining attention to favorable coping strategies are important neurocognitive processes for effective coping. Executive dysfunction, or comorbid problems associated with executive dysfunction, may thus augment pain processing and impede the utilization of coping strategies. Together, this suggests that ACT may be particularly beneficial for chronic pain patients high in autism traits and/or ADHD symptomatology, partially due to its ability to improve psychological flexibility, which is regarded a component of executive functioning. However, the impact of such comorbid neurodevelopmental disorders in pediatric chronic pain on treatment outcome is yet unknown. Despite that acceptance-based therapies demonstrate promising results for improving socioemotional functioning and pain interference in chronic pain, considerable interpatient variability in the response to ACT exists (e.g., Hann and McCracken, 2014). Insight into what works for whom is crucial for patient–treatment matching to maximize treatment outcome and thereby limit the impact of chronic pain and comorbid problems on the patient's emotional, cognitive, and physical development (Vlaeyen and Morley, 2005; Edwards et al., 2016).

To this end, the primary aim of the current single-arm trial was to assess the association between comorbid neuropsychiatric symptoms (i.e., autism traits and ADHD symptoms) in pediatric chronic pain patients and (1) pre-ACT functioning and (2) change from pre- to post-ACT. Outcomes were defined as the degree to which pain interfered with daily activities (i.e., pain interference, sleep, school and physical functioning), socioemotional functioning (i.e., depressive symptoms, emotional and social functioning), psychological inflexibility, and pain intensity.

## Methods

### Treatment

The intervention consisted of a standard face-to-face ACT-based treatment. The treatment consisted of 17 sessions involving four phases with different but interrelated treatment objectives: (1) preparing for behavioural change (sessions 1–3), (2) shifting perspective (sessions 4–6), (3) acceptance and cognitive defusion (sessions 7 and 8), and (4) values-oriented behavioral activation (sessions 9–17). In every session, participants were given individualized home assignments related to the treatment content and to their own specific challenges, and these outcomes were discussed at the beginning of the following session. Five ACT-trained psychologists delivered the treatment. All psychologists were continuously supervised by a senior researcher with extensive experience using ACT for pediatric chronic pain. To promote a uniform approach among the treatment providers, patients were discussed with all therapists during clinical supervision meetings. No other therapist monitoring measures were utilized. For a detailed description of the ACT protocol, see Kanstrup et al. (2016). A parent support program was embedded in the treatment and comprised four parent sessions and one joint session including the parents and the patient. The objective of the parent program was to enhance the parents' ability to support their child to improve functioning, by means of pain education, contingency management including clarification of own values, and the use of acceptance skills to manage their own distress due to their child's pain. For the purpose of the present study, a single group of patients in which all received active treatment was studied.

### Participants

Participants in this convenience sample were 47 children and adolescents (8–18 years old, 33 girls) with chronic pain, recruited via a tertiary pain clinic. Patients referred to the clinic because of chronic debilitating pain were considered eligible for the study if they (1) suffered from pain for more than 6 months, (2) reported insufficient effects of previous pain treatments, and (3) reported substantial pain-related disability. Patients were not considered as eligible if they had psychiatric comorbidity that required immediate intervention; substantial risk for suicide or substantial cognitive dysfunction; insufficient proficiency in Swedish; other ongoing or planned treatments (i.e., within the next 6 months); and pain that was fully explained by a pathophysiological process, e.g., cancer. The study was approved by the ethical review board in Stockholm, Sweden. All participants (parents and children) provided written informed consent to participate in the study.

### Assessment

Data collection took place pre- and post-ACT. Eligibility was assessed in a semistructured clinical interview during the first visit. Patient's functioning was assessed through patient- and parent-reported questionnaires (see below).

### Measures

Questionnaires were completed by the children, except for the Social Responsiveness Scale (SRS) and the Conners Third Edition (Conners-3). The following questionnaires were completed at pretreatment and posttreatment, as part of a clinical routine using paper and pencil.

#### Pain Interference

The six-item Pain Interference Index (PII) was used to assess the influence of pain on behavior or to what extent pain impacts everyday functioning (e.g., schoolwork, leisure activities, sleep). Items are rated on a scale from 0 (not at all) to 6 (completely), and the maximum score is 36. A higher score indicates more pain interference. The Swedish version of PII has shown sensitivity to change (Wicksell et al., 2010). The Cronbach α was 0.83.

#### Insomnia Severity

The Insomnia Severity Index (ISI) is a seven-item measure that assesses the individual's subjective perception of sleep complaints, rated on a five-point Likert scale and a maximum score of 28. A higher score suggests more severe insomnia (Bastien et al., 2001). Internal consistency was high in the current sample (Cronbach α = 0.87).

#### Quality of Life

The Pediatric Quality of Life Inventory (PedsQL) is a 23-item measure that evaluates the child's quality of life in four areas of functioning: physical functioning (eight items), emotional functioning (five items), social functioning (five items), and school functioning (five items). Two age versions were used; one for children 8–12 years old and one for adolescents 13–18 years old. Cronbach α's for the respective subscales ranged from 0.66 to 0.86. The instrument uses a five-point Likert scale from 0 (never a problem) to 4 (almost always a problem) to indicate severity. A higher score indicates better functioning (maximum score is 100) (Bastiaansen et al., 2004).

#### Depression

The Center for Epidemiological Studies–Depression Scale Children (CES-DC) (Faulstich et al., 1986) consists of 20 items concerning feelings and actions relevant for depressive disorder, rated on a scale from 0 (not at all) to 4 (a lot). The maximum score is 60. Higher scores indicate higher levels of depression. CES-DC has shown adequate psychometric properties and the ability to discriminate depressive disorder in a Swedish population of adolescents (Olsson, 1997). The overall Cronbach α was 0.80.

#### Psychological Inflexibility

The Psychological Inflexibility in Pain Scale (PIPS) is a measure of psychological inflexibility, measuring avoidance of pain and fusion with pain thoughts (Wicksell et al., 2010). The PIPS consists of 12 items rated on a scale from 1 (never true) to 7 (always true), with scores ranging from 12 to a maximum of 84. Higher scores indicate greater levels of psychological inflexibility. Internal consistency (Cronbach α) of the total scale was 0.89 in the current sample.

#### Pain Intensity

The Lubeck Pain-Screening Questionnaire (LPQ) evaluates the prevalence and consequences of pain during the previous 3 months (Roth-Isigkeit et al., 2004). As single items of the LPQ have been shown to be valid and reliable measures of pain, the current study used “How strong is your main pain usually?” to construct a pain intensity variable. The item was rated on a visual analog scale (VAS) from 0 (“hardly noticeable pain”) to 100 (“strongest imaginable pain”).

The following questionnaires were completed at pretreatment only, as part of a clinical routine using paper and pencil.

#### Autism Traits

The SRS-Parent report is a 65-item parent report for children and adolescents (4–18 years of age) that measures the severity of autism spectrum symptoms. Scoring is on a four-point Likert scale. Higher scores indicate a higher degree of social impairment. SRS scores of 60 T or higher indicate a level of autistic social impairment that is clinically significant (Constantino and Gruber, 2014). *T* scores are gender-corrected scores (gender of the child). Standardized *T* scores with mean = 50 (SD = 10) were used in the current study. Internal consistency (Cronbach α) of the total scale is 0.94 (parent rated) (Constantino and Gruber, 2005).

#### Attention-Deficit/Hyperactivity Disorder

The Conners-3 ADHD Index 10-item subscale (i.e., Conners Hyperactivity Index) of the Conners 3rd Edition–Parent (Conners-3-P, 110 items) was used to obtain the parent's observation about the most prominent symptoms of ADHD over the last month in their child (Conners, 2008). This 10-item scale is recommended as a quick and valid research and clinical screening tool in large samples. It has been shown to accurately differentiate children with ADHD from those without the clinical diagnosis (Chang et al., 2016). Items are based on *Diagnostic and Statistical Manual of Mental Disorders, Fifth Edition* criteria and scored on a four-point Likert scale. Standardized *T* scores with mean = 50 (SD = 10) were used in the current study. *T* scores are gender- and age-corrected scores. A higher score indicates more symptoms. ADHD scores of 65 T or greater indicate a level of ADHD that is clinically significant. Internal consistency (Cronbach α) of the Conners-3 Hyperactivity Index is 0.94 (parent rated) (Kumar and Steer, 2003).

#### Duration and Frequency of Pain

Two items of the LPQ were used to assess frequency of pain (“How often do you have this pain?”) and duration of pain (“How long have you had pain?”) (Roth-Isigkeit et al., 2004). Each item was rated on a five-point scale. As indicated earlier, pain intensity was assessed pre- and post-ACT on a VAS.

### Sample Size

Based on previous studies of ACT in chronic pain patient groups, medium (*d* = 0.6) to large (*d* = 0.8) within-group effect sizes were expected for pain interference and pain intensity. A small to medium effect size was expected for depression (Wicksell et al., 2007, 2009; Gauntlett-Gilbert et al., 2013). Power analysis using a power of 80% and an α of 0.05 suggests that a total sample between 15 and 34 is adequate to detect medium to large effect sizes. As attrition was expected and to take into account the possibility of small to medium effect sizes, we aimed to recruit a minimum of 45 patients.

### Statistical Analysis

Analyses were conducted in a series of steps in SPSS version 24 (SPSS Inc., Chicago, IL, USA) and JASP (version 0.13, JASP Team, 2020). Means and standard deviations were calculated for descriptive purposes. Correlational analyses were used to assess the relationship of pretreatment functioning with level of autistic traits and with ADHD symptoms. Mixed models were used to estimate the change from pre- to post-ACT for pain interfering with daily activities (insomnia; ISI), school and physical functioning (subscales of the PedsQL), pain interference (PII), socioemotional functioning (depressive symptoms; CES-D), emotional and social functioning (subscales of the PedsQL), psychological inflexibility (PIPS), and pain intensity (item of LPQ). Then, mixed-model regressions were conducted for autistic traits (SRS) and ADHD symptoms (Conners-3-P) separately to assess associations between autism/ADHD and ACT outcomes. Model simplicity and likelihood ratio tests were used to select appropriate covariance structures. Data for the main measures were analyzed using timepoint (pretreatment and posttreatment) as a repeated and fixed factor and subject as a random factor. Continuous autism/ADHD *T* scores were added as a fixed factor. The effects of interest were main effects of time and autism/ADHD and interaction effects of time × autism/ADHD. Subsequently, each model with a significant interaction effect controlled for gender and age (fixed factors) (results are shown in the Supplementary Materials). Variables were *Z*-transformed before analysis yielding standardized regression coefficients. The pre- to post-ACT changes analyses were repeated with a between-subjects factor, dividing patients into above or below clinically significant levels of autism traits and/or ADHD symptoms based on the criteria for clinically significant *T* scores (i.e., 60 T or higher for autism traits, 65 T or higher for ADHD) (results are shown in Supplementary Materials). Alpha Values were set at 0.05 throughout. In addition to traditional null hypothesis significance testing, Bayes factors (BF_10_) were calculated using Bayesian correlation analyses with default prior probabilities. Bayes factors provide relative evidence of both the null (H0) and alternative (HA) hypothesis, compared to the conclusions about the null hypothesis proffered by traditional null hypothesis significance testing.

## Results

### Sample Characteristics

Sample characteristics are presented in Table 1. Data on autistic traits and ADHD symptoms were available for 38 and 40 patients, respectively. See also Supplementary Table 1 for an overview of the missing variables separately for pre- and post-ACT. No information regarding reasons for attrition was obtained. Clinically significant levels of autism traits and ADHD symptoms occurred in 13.2% (*n* = 5) and 22.5% (*n* = 9) of the patients, respectively. Ten percent (*n* = 4) of the patients scored greater than clinically significant levels for both autism traits and ADHD symptoms. The current data are based on partially the same sample as was used in Lipsker et al. (2018).

**Table 1:** Descriptive characteristics of the study population.

**Characteristic**	
N	47
**Age (years) (*****n*** **=, 47)**	
Mean (SD)	14.8 (2.2)
Range	9.5–17.9
Gender (% girls)	70
**Pain characteristics**	
Pain duration	
Every now and then (%)	0
1–3 months (%)	2.1
<1 month (%)	0
>3 months (%)	6.4
>6 months (%)	17.0
>12 months (%)	53.2
Pain frequency	
<1 × per month (%)	0
1 × per month (%)	0
2–3 × per month (%)	4.3
1 × per week (%)	4.3
Multiple times per week (%)	14.9
Every day	55.3
Pain intensity (0–100) (SD)	57.2 (13.1)
**Autistic traits (SRS) (*****n*** **=, 38)**	
Mean (SD)	47.3 (8.8)
Range	34–71
Clinically significant level (%)	13.2
**ADHD symptoms (Conners-3) (*****n*** **=, 40)**	
Mean (SD)	55.3 (12.6)
Range	46–89
Clinically significant level (%)	22.5

### Relationships Between Autism/ADHD and Pretreatment Functioning

#### Autism

As shown in Table 2, at pretreatment, patients with higher levels of autism reported greater pain interference, higher depression, greater psychological inflexibility, and lower physical and social functioning, although the latter two were non-significant. Bayesian statistics revealed anecdotal (Bayes factor 1–3), moderate (Bayes factor 3–10), and strong (Bayes factor 10–30) evidence in favor of the alternative relative to the null hypothesis (Wagenmakers et al., 2017).

**Table 2:** Pearson correlation coefficients, 95% confidence intervals (CIs), and Bayes factors (BF_10_) of each pretreatment outcome with autism traits and with ADHD symptoms.

	**Autism traits**			**ADHD symptoms**		
	***r***	**BF_**10**_**	**95% CI**	***r***	**BF_**10**_**	**95% CI**
Pain interference	0.430^**^	6.67	0.10–0.64	0.377^*^	2.80	0.07–0.62
Physical functioning	−0.298^#^	0.99	−0.56–0.02	−0.211	0.44	−0.50–0.12
School functioning	−0.180	0.36	−0.47–0.15	−0.271	0.74	−0.54–0.05
Insomnia	0.120	0.26	−0.22–0.44	0.097	0.24	−0.24–0.42
Depression	0.406^*^	4.43	0.10–0.64	0.342^*^	1.43	0.03–0.60
Emotional functioning	−0.249	0.60	−0.53–0.08	−0.413^*^	4.95	−0.65−−0.11
Social functioning	−0.313^#^	1.18	−0.58–0.01	−0.256	0.64	−0.53–0.07
Psychological inflexibility	0.520^***^	38.48	0.24–0.72	0.291	0.92	0.01–0.60
Pain intensity	0.109	0.25	−0.23–0.42	−0.022	0.21	−0.35–0.31
Pain frequency	−0.001	0.21	−0.33–0.32	−0.038	0.21	−0.36–0.29
Pain duration	−0.130	0.27	−0.44–0.29	−0.624^***^	723.41	−0.79−−0.38

*** *p < 0.001*,

** *p < 0.01*,

* *p < 0.05*,

# *p < 0.07*.

#### ADHD

**Figure 1:**
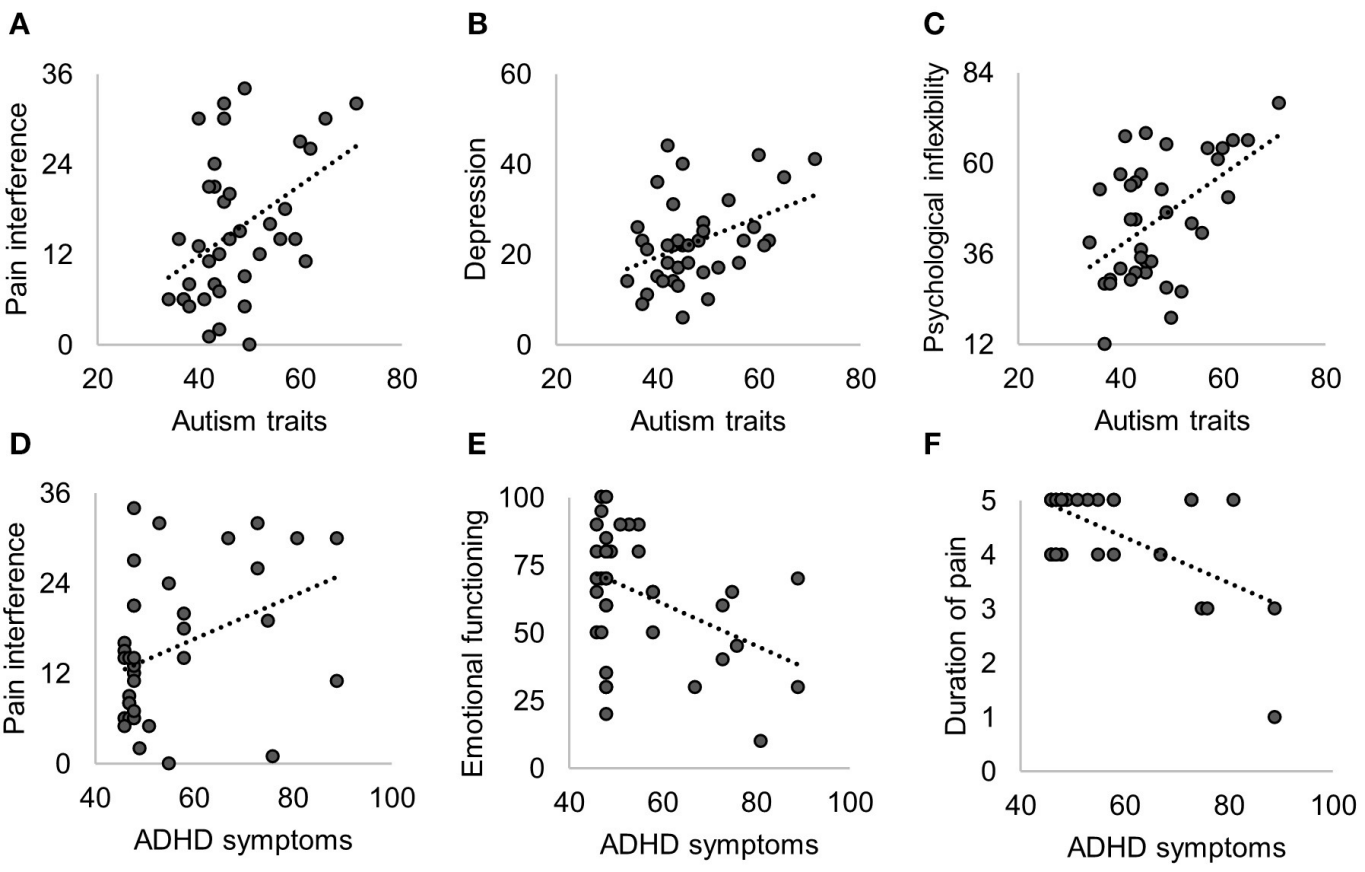
Significant Pearson correlations of pretreatment functioning with autism traits **(A–C)** and with ADHD symptoms **(D–F)**. Non-significant correlations are shown in Table 2 and Supplementary Figure 1.

At pretreatment, patients with higher levels of ADHD symptoms reported greater pain interference, higher depression, lower emotional functioning, and longer duration of pain. The significant relationships between autism/ADHD and pretreatment functioning are displayed in Figure 1. An overview of all correlation analyses results (both statistically significant and non-significant) and the corresponding Bayes factors are shown in Table 2. All non-significant correlation plots are shown in Supplementary Figure 1.

### Pre- and Post-act Comparisons

**Figure 2:**
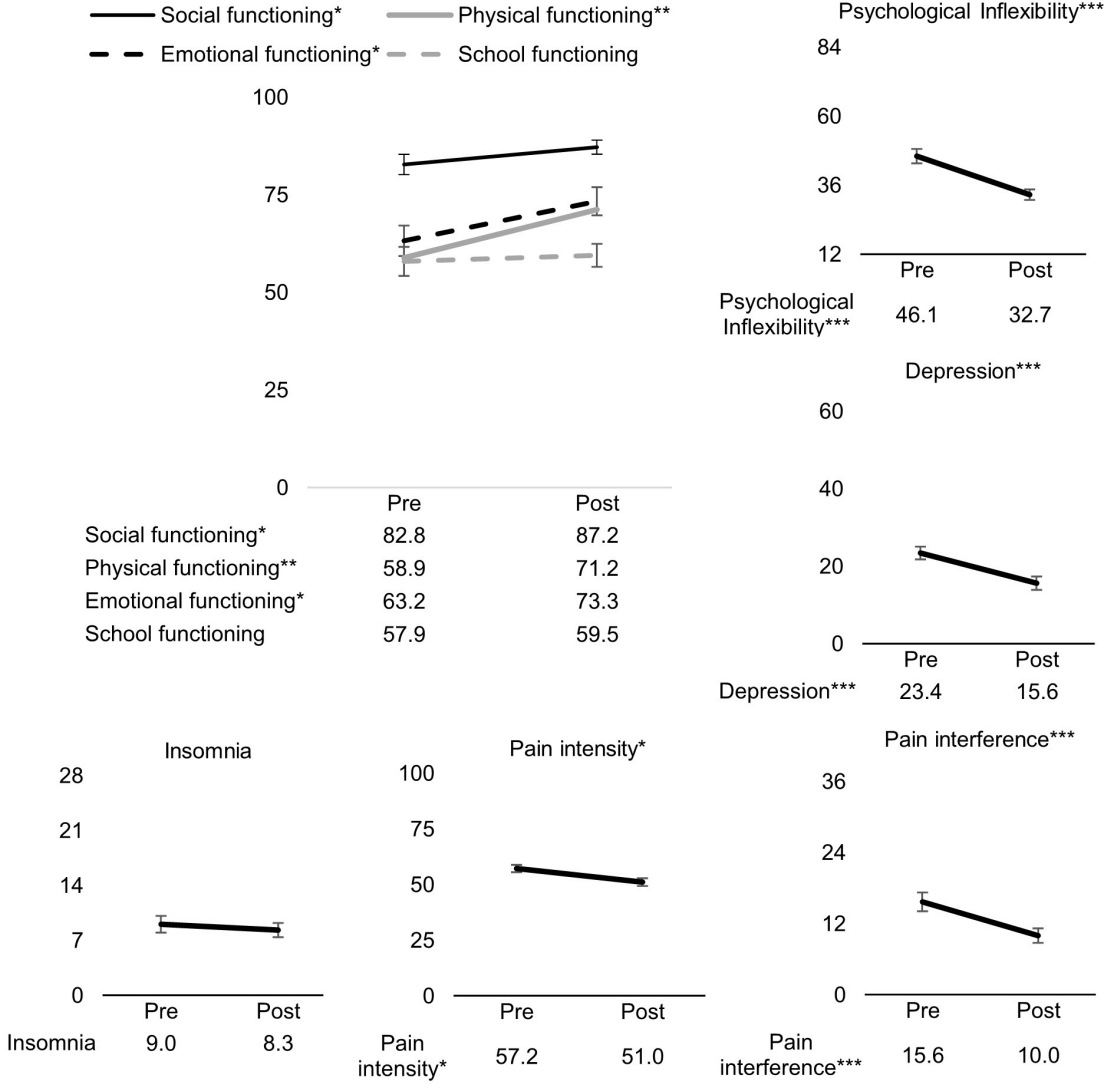
Pre- to post-ACT changes for all outcome measures. Values on the *y*-axes are mean scores pre- and post-ACT. Errors bars represent standard error of the mean. ***p < 0.001, **p < 0.01, *p < 0.001.

As can been seen in Figure 2, statistically significant improvements from pre- to post-ACT were found for pain interference [*t*_(1,41)_ = −4.13, *β* = −0.69, 95% confidence interval (CI) [−1.03, −0.35], *p* < 0.001], physical functioning [*t*_(1,41)_ = 3.42, *β* = 0.57, 95% CI [0.23, 0.91], *p* = 0.001], depression [*t*_(1,44)_ = −4.46, *β* = −0.65, 95% CI [−0.95, −0.36], *p* < 0.001], emotional functioning [*t*_(1,44)_ = 2.70, *β* = 0.35, 95% CI [0.09, 0.61], *p* = 0.010], social functioning [*t*_(1,38)_ = 2.45, *β* = 0.42, 95% CI [0.07, 0.76], *p* = 0.019], psychological inflexibility [*t*_(1,42)_ = −5.13, *β* = −0.83, 95% CI [−1.16, −0.50], *p* < 0.001], and pain intensity [*t*_(1,43)_ = −2.50, *β* = −0.45, 95% CI [−0.82, −0.09], *p* = 0.016]. Insomnia and school functioning did not significantly change from pre- to post-ACT.

### Associations Between Autism Traits/ADHD Symptoms and Treatment Outcome

#### Autism

**Figure 3:**
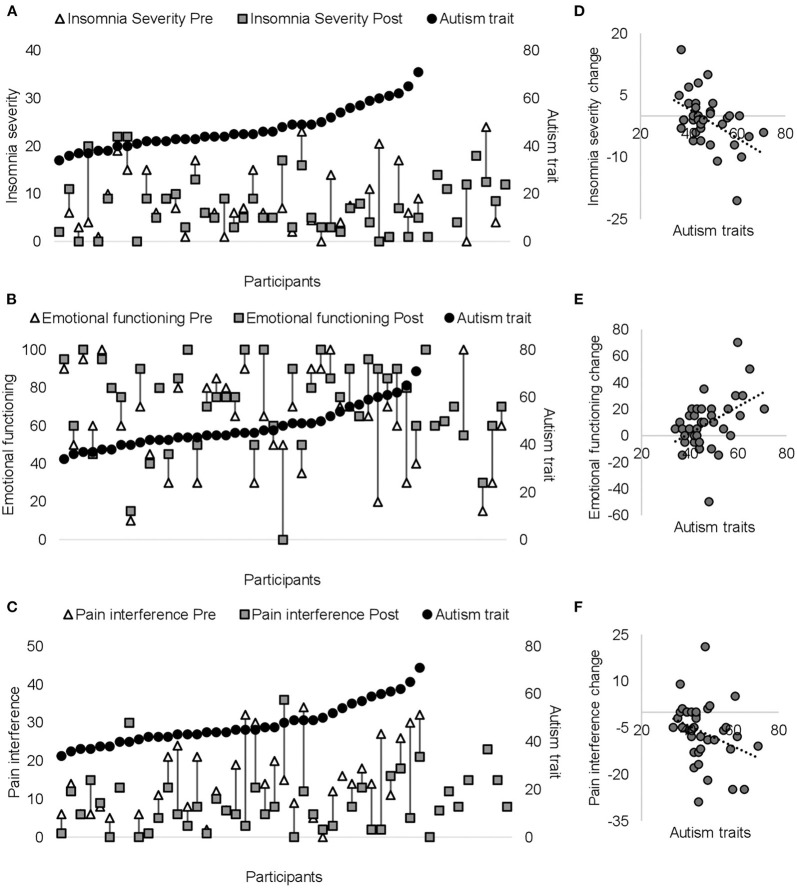
Pre- (white triangles) and post-ACT scores (dark gray squares) are plotted on the primary (left) *y*-axis for each patient for insomnia **(A)**, emotional functioning **(B)**, and pain interference **(C)**. Black circles on secondary *y*-axis represent individual autism scores (presented from the lowest to the highest score). The right panel **(D–F)** shows significant correlations between autism traits and change score (from pre- to post-ACT) for insomnia **(D)** emotional functioning **(E)**, and pain interference **(F)**. Negative insomnia severity/pain interference change scores indicate greater improvements in insomnia/pain interference. Positive emotional functioning change scores indicate greater improvements in emotional functioning.

As shown in Figure 3 and Table 3, significant time × autism interactions were evident for insomnia severity and emotional functioning, indicating that patients higher in autism trait showed greater improvements in insomnia and emotional functioning. Similarly, those higher in autism trait showed greater improvements in pain interference, although non-significant. Controlling for gender and age did not alter the direction of the uncorrected results (see Supplementary Materials for age- and gender-adjusted results). Similar results were found when patients were divided into below and above clinically significant levels of autism or ADHD (see Supplementary Figure 2).

**Table 3:** Results of the mixed regression analyses of autism traits/ADHD symptoms and change from pre- to post-ACT for all outcomes (autism/ADHD*time); 95% CI = 95% confidence interval; ***p < 0.001, **p < 0.01, *p < 0.05, ^#^*p* < 0.07.

	**Autism traits**			**ADHD symptoms**		
	***β***	***t***	**95% CI**	***β***	***t***	**95% CI**
Pain interference	−0.32^#^	−1.96	−0.65–0.01	−0.15	−0.89	−0.49–0.19
Physical functioning	0.10	0.69	−0.19–0.38	0.05	−0.32	−0.24–0.33
School functioning	−0.08	0.68	−0.16–0.33	0.07	0.59	−0.17–0.32
Insomnia	−0.46**	−3.01	−0.76−−0.15	−0.14	−0.84	−0.48–0.20
Depression	−0.24	−1.70	−0.53–0.05	0.06	0.41	−0.24–0.35
Emotional functioning	0.36**	3.09	0.12–0.60	0.22	1.76	−0.03–0.48
Social functioning	−0.09	0.54	−0.25–0.44	−0.01	−0.06	−0.36–0.33
Psychological inflexibility	−0.21	−1.30	−0.54–0.12	0.01	0.06	−0.33–0.36
Pain intensity	−0.05	−0.34	−0.37–0.27	0.03	0.19	−0.29–0.35

### ADHD

No significant time × ADHD interactions were evident (see Table 3 for an overview of all results).

## Discussion

In the current study, pediatric chronic pain patients showed improvements from pre- to post-ACT in interference of pain with daily activities, socioemotional functioning, psychological inflexibility, and pain intensity, contributing to the growing body of evidence showing that acceptance-based treatments improve functioning in pediatric chronic pain. Those with greater neuropsychiatric traits and symptoms (autism and ADHD) benefited at least to the same degree from ACT as compared to those lower in neuropsychiatric traits and symptoms. In fact, those higher in autism traits showed greater improvements in insomnia and emotional functioning. However, because of the lack of a control treatment, it cannot be concluded that improvements resulted from ACT, as they might have arisen from non-specific effects of ACT, natural improvements placebo effects, or because of changes in patient characteristics. Nevertheless, clinical improvements of such a large extent are unlikely to be spontaneous. Moreover, improvements were consistent with the ACT model and consonant with previous evidence (e.g., Hann and McCracken, 2014; Veehof et al., 2016; Hughes et al., 2017), in that improvements in psychological inflexibility, pain interference, and functioning were achieved.

Greater improvements in insomnia and emotional functioning were seen in those with greater levels of autism. It has been proposed that pediatric chronic pain can disrupt sleep, which can, independently of pain, promote physiological and psychological changes that exacerbate pain, suggesting that pain and sleep operate in a bidirectional manner (Lewin and Dahl, 1999; Valrie et al., 2013). Besides that sleep disturbances are commonly reported by pediatric chronic pain patients (Valrie et al., 2013), such disturbances are also a prevailing problem in autism and ADHD (Cortese et al., 2009; Elrod and Hood, 2015). Considering that poor sleep compromises the emotional, cognitive, and behavioral development of adolescents with chronic pain (Palermo and Kiska, 2005), improvements of sleep in this young chronic pain population with greater autism traits are highly encouraging. Greater autism traits were further associated with lower pre-ACT functioning, highlighting the possibility that there was a greater room for improvement in those with greater autism traits. However, this unlikely explains the added benefit with higher autism traits as insomnia and emotional functioning were not associated with autism traits before ACT. Keeping the behavioral problems associated with autism in mind, such as deficits in executive functions and associated social challenges (Weiss et al., 2018), a perhaps more reasonable explanation for the positive association between autism traits and treatment outcome is that these individuals in particular may benefit from the structure that a behavioral-based treatment provides as well as ACT's methodology to increase awareness and understanding of one's own thoughts and feelings. In speculation, such treatment may provide help in directing attentional resources to adaptive behaviors.

Greater improvement in selective symptoms was not seen in patients higher in ADHD symptoms. In part similar to autism, the level of ADHD was associated with lower pre-ACT functioning in some domains (i.e., pain interference, depression, emotional functioning, and duration of pain), but this did not lead to greater improvements after treatment. Despite that autism and ADHD depend in part on a shared neural dysfunction and often co-occur (Ghirardi et al., 2018; Kernbach et al., 2018), autism and ADHD show considerable differences in symptom profiles, and autistic traits are not common in ADHD (Mayes et al., 2012). For example, children with autism show more prominent deficits in cognitive flexibility as compared to children with ADHD (Corbett et al., 2009). A similar pattern was observed in the current study, in which psychological flexibility (which overlaps with cognitive flexibility; Whiting et al., 2017) was associated with autism traits only. Although speculative at this point, improvements in psychological flexibility, the core target of ACT, enabled those higher in autism traits to cope better with negative private experiences such as pain and distress and gain more from the treatment (Compas et al., 2017). ACT may have potentially also improved specific autism-related traits. Indeed, ACT improved prosocial behavior and emotional distress in a clinical sample of children with autism spectrum disorder (Pahnke et al., 2014), suggesting that ACT may positively affect behaviors connected to autistic traits. Considering that functioning improved from pretreatment to posttreatment in not only those low but also those high in autism traits and ADHD symptoms, ACT could potentially be a suitable treatment for improving functioning in individuals with such neuropsychiatric traits.

The current results need to be seen in the light of several limitations. First, lack of a control group receiving a comparator (e.g., physical therapy) or no (e.g., waiting list) treatment can be considered as a major limitation, as pre- to post-ACT changes may be due to specific treatment components but could also reflect natural improvement of functioning, expectation (because the patients and their parents could not be blinded), or developmental changes in the patient due to becoming older. Second, this study was conducted with a limited sample size, and null findings should thus be interpreted with some caution. In particular, the number of patients above cutoff for clinically significant autism (*n* = 5) or ADHD (*n* = 9) is low. Thus, also positive findings should be interpreted with caution, and the generalizability of the findings to clinical samples of autism and ADHD remains unknown until larger controlled studies using diagnostic testing have been performed. Future controlled clinical trials may also monitor treatment fidelity. Third, findings from this study, and related studies (Lipsker et al., 2018; Low Kapalu et al., 2018), suggest that the combination of pediatric chronic pain and neurodevelopmental issues is common and presents significant challenges to the child and his/her family. Future research could evaluate the role of neurodevelopmental symptoms in aspects such as dropout and treatment adherence. Fourth, ACT was explicitly aimed at creating sustained changes, and even though improvements were observed directly post-ACT, whether these persist long-term, in addition to the specificity of ACT, requires further examination. Future studies may address these limitations by conducting larger controlled studies including chronic pain patients as well as patients diagnosed with autism and/or ADHD. Addressing whether severity of autism traits and ADHD symptoms can be reduced in response to ACT as well as which factors enabled chronic pain patients, and specifically those higher in autism trait, to gain from ACT can provide mechanistic links connecting improved functioning with ACT. We suggest here two potential factors that deserve further scrutiny: psychological flexibility and attention to interoceptive cues. First, as discussed previously, improvements in psychological flexibility, the core target of ACT, may enable greater gains from the ACT sessions. Intermediate assessment of psychological flexibility may shed light onto the time course of improvement and the possibility to gain more from each ACT session. Second, it has been suggested that autism patients disproportionally allocate attention recourses to internal rather than to external cues (Schauder et al., 2015). Whether a shift in attention away from interoceptive cues and sustaining attention toward helpful coping strategies can boost patients' ability to gain more from the treatment is a question that future research may consider.

Taken together, even though the results should be viewed in light of its limitations, the results of the current study suggest that pediatric chronic pain patients higher in autistic traits could have an extra benefit from ACT and those higher in ADHD symptomology benefit to the same degree as those low in ADHD symptomology.

## Data Availability Statement

The dataset of this article is accessible on request from the corresponding author.

## Ethics Statement

The studies involving human participants were reviewed and approved by Regional ethical review board in Stockholm: 2013/231-31-4. Written informed consent to participate in this study was provided by both the participant and the participants' legal guardian/next of kin.

## Author Contributions

CW, RW, and ML conceived the experiment. CW data collection. LB statistical analysis. LB, CW, RW, and ML writing. All authors reviewed the manuscript.

## Conflict of Interest

The authors declare that the research was conducted in the absence of any commercial or financial relationships that could be construed as a potential conflict of interest.
