# Supplementary material for: Neuropsychiatric Symptoms in Pediatric Chronic Pain and Outcome of Acceptance and Commitment Therapy
Source: Front Psychol. 2021 Apr 9;12:576943. doi: 10.3389/fpsyg.2021.576943 (PMC8062759; doi:10.3389/fpsyg.2021.576943)
Supplement: Supplementary file 1 [file Data_Sheet_1.pdf]

Supplementary Material

Table S1. Available sample size for each variable, for pre-ACT and post-ACT measurements; / indicates only administered pre-ACT.

	Pre-ACT *n*	Post-ACT *n*
Sex	47	47
Age	47	47
Pain Interference (PII)	38	46
Insomnia Severity (ISI)	38	47
Physical functioning	43	46
Emotional functioning	42	46
Social functioning	38	46
School functioning	46	45
Depression (CES-DC)	38	42
Psychological Inflexibility (PIPS)	41	45
Pain intensity (LPQ)	39	44
Autism traits (SRS-Parent)	38	/
Attention Deficit/Hyperactivity Disorder (subscale of Conners 3-Parent)	40	/
Duration of pain (LPQ)	37	/
Frequency of pain (LPQ)	37	/

1 Age- and sex-adjusted associations between autism traits/ADHD symptoms and ACT outcome

Autism traits. The significant time x autism interactions for insomnia and emotional functioning remained significant when adjusting for age and sex (insomnia (t(1, 36) = -3.10, ß = -0.47, 95% CI [-0.78, -0.16], p = .004; emotional functioning (t(1, 38) = 3.09, ß = 0.36, 95% CI [0.12, 0.60], p = .004)). The non-significant time x pain interference interaction remained unchanged when adjusting for age and sex (t(1, 38) = -1.96, ß = -0.32, 95% CI [-0.65, 0.01], p = .057).

2 Clinically significant levels autism traits/ADHD symptoms and ACT outcomes

As can be seen in Figure S2, between-group analysis showed that those with clinically significant levels of autism traits and/or ADHD symptoms improved more relative to those without clinically significant levels (insomnia (t(1, 35) = -2.53, ß = -0.85, 95% CI [-1.53, -0.17], p = .016); emotional functioning t(1, 38) = 2.87, ß = 0.93, 95% CI [0.27, 1.58], p = .007)). Pre- to post-treatment pain interference improvement did not significantly differ between low and high clinically significant levels autism traits/ADHD symptoms (t(1, 38) = -1.07, ß = -0.40, 95% CI [-1.15, -0.36], p = .293). Please note that samples are small (n = 9 above clinically significant level and n = 29 below clinically significant level).

3. Supplementary Figures

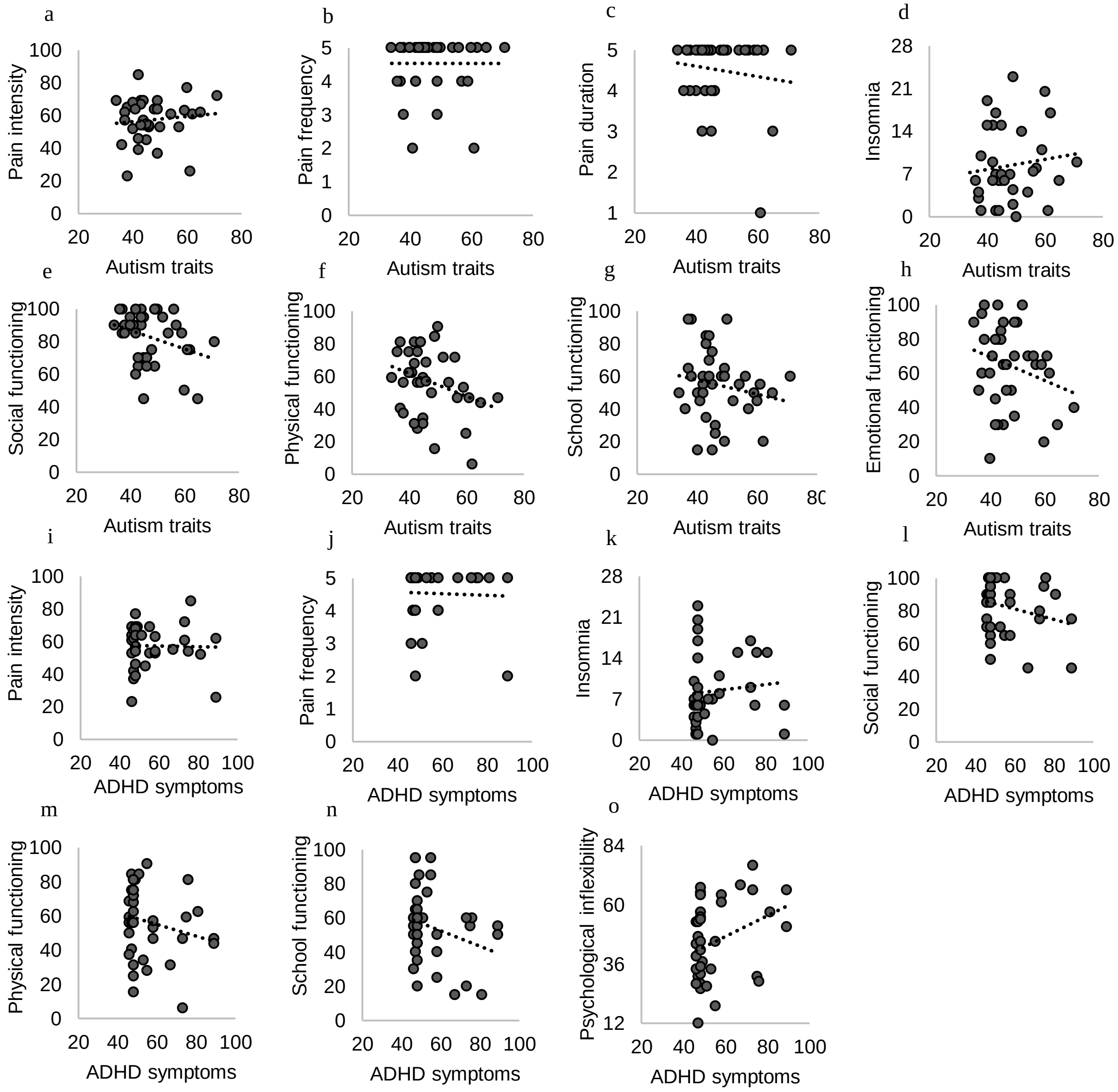

Figure S1. Pearson correlation plots of outcome measures with autism traits (a-h) and with ADHD (i-o) symptoms, showing all statistically non-significant correlations.

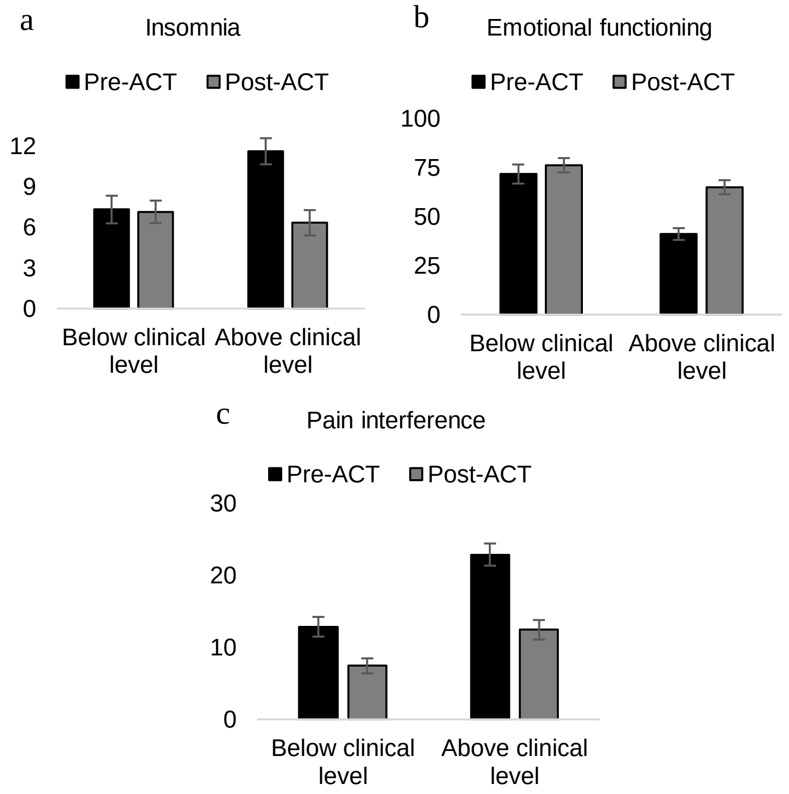

Figure S2. Average pre- (black) and post-ACT scores (gray) for those below (n = 29) and above (n = 9) clinically significant levels of autism traits and/or ADHD symptoms, plotted for insomnia (a), emotional functioning (b), and pain interference (c). Errors bars represent standard error of the mean.
